# Biliary obstruction and pancreatitis after duodenal stent placement in the descending duodenum: a retrospective study

**DOI:** 10.1186/s12876-022-02333-7

**Published:** 2022-05-21

**Authors:** Junichi Kaneko, Hirotoshi Ishiwatari, Koiku Asakura, Tatsunori Satoh, Junya Sato, Kazuma Ishikawa, Hiroyuki Matsubayashi, Yohei Yabuuchi, Yoshihiro Kishida, Masao Yoshida, Sayo Ito, Noboru Kawata, Kenichiro Imai, Kohei Takizawa, Kinichi Hotta, Hiroyuki Ono

**Affiliations:** 1 Division of Endoscopy, Shizuoka Cancer Center, 1007 Shimonagakubo Nagaizumi-cho, Sunto-gun, Shizuoka, 411-8777 Japan; 2 Division of Diagnostic Radiology, Shizuoka Cancer Center, Shizuoka, Japan; 3 Division of Genetic Medicine Promotion, Shizuoka Cancer Center, Shizuoka, Japan

**Keywords:** Gastric outlet obstruction, Self-expandable metallic stent, Duodenal stent, Biliary obstruction, Pancreatitis

## Abstract

**Background:**

Metallic stents placed in the descending duodenum can cause compression of the major duodenal papilla, resulting in biliary obstruction and pancreatitis. These are notable early adverse events of duodenal stent placement; however, they have been rarely examined. This study aimed to assess the incidence of and risk factors for biliary obstruction and/or pancreatitis after duodenal stent placement in the descending duodenum.

**Methods:**

We retrospectively reviewed data of consecutive patients who underwent metallic stent placement in the descending duodenum for malignant gastric outlet obstruction at a tertiary referral cancer center between April 2014 and December 2019. Risk factors for biliary obstruction and/or pancreatitis were analyzed using a logistic regression model.

**Results:**

Sixty-five patients were included. Biliary obstruction and/or pancreatitis occurred in 12 patients (18%): 8 with biliary obstruction, 2 with pancreatitis, and 2 with both biliary obstruction and pancreatitis. Multivariate analysis indicated that female sex (odds ratio: 9.2, 95% confidence interval: 1.4–58.6, *P* = 0.02), absence of biliary stents (odds ratio: 12.9, 95% confidence interval: 1.8–90.2, *P* = 0.01), and tumor invasion to the major duodenal papilla (odds ratio: 25.8, 95% confidence interval: 2.0–340.0, *P* = 0.01) were significant independent risk factors for biliary obstruction and/or pancreatitis.

**Conclusions:**

The incidence of biliary obstruction and/or pancreatitis after duodenal stent placement in the descending duodenum was non-negligible. Female sex, absence of biliary stents, and tumor invasion to the major duodenal papilla were the primary risk factors. Risk stratification can allow endoscopists to better identify patients at significant risk and permit detailed informed consent.

**Supplementary Information:**

The online version contains supplementary material available at 10.1186/s12876-022-02333-7.

## Background

Malignant gastric outlet obstruction (mGOO) caused by several advanced cancers leads to nausea, vomiting, and food intake intolerance, resulting in deterioration of quality of life. Surgical gastrojejunostomy and endoscopic duodenal stent placement (DSP) using self-expandable metallic stents (SEMSs) are widely utilized to relieve symptoms of mGOO. Several previous studies have demonstrated that DSP allows for earlier resumption of food intake, a shorter hospitalization period, and earlier administration of chemotherapy after interventions relative to surgical gastrojejunostomy [1–5]. Thus, DSP has recently become more common than surgical gastrojejunostomy. However, several early adverse events (AEs) of DSP have been reported, including bleeding, perforation, obstruction, stent migration, biliary obstruction, and pancreatitis [1–15].

Of these, biliary obstruction and pancreatitis can be caused by compression of the major duodenal papilla (MDP) due to SEMSs [11, 16, 17]. Thus, biliary obstruction and pancreatitis can develop only after DSP in the descending duodenum. A previous retrospective study reported that 11% of patients (9/94) developed pancreatitis after DSP in the descending duodenum and that SEMS bridging the MDP can cause pancreatitis [11]. In contrast, few studies have investigated biliary obstruction after DSP in the descending duodenum. Biliary obstruction and/or pancreatitis after DSP (BPAD) is a notable early AE because it may require additional treatment, delay chemotherapy, and prolong the duration of hospitalization; however, few studies have focused on BPAD. Accordingly, this study aimed to assess the incidence of and risk factors for BPAD.

## Methods

### Patient

In this retrospective study, we reviewed data of consecutive patients who underwent their first SEMS placement in the duodenum, including the descending duodenum, at the Shizuoka Cancer Center between April 2014 and December 2019. The exclusion criteria were absence of computed tomography (CT) imaging within 1 month before DSP and the presence of percutaneous or transmural biliary drainage before DSP. All patients provided written informed consent for DSP, and the study was approved by the institutional review board (IRB) of Shizuoka Cancer Center (IRB number: J2019-174–2019-1). All investigations were performed in accordance with the ethical standards of the Declaration of Helsinki.

### Duodenal stent placement

DSP was performed with the patient under conscious sedation. An endoscope (GIF 1 T-240, PCF 240 or JF 260 V; Olympus, Tokyo, Japan) and an endoscopic retrograde cholangiopancreatography (ERCP) catheter with a biliary guidewire were used. Uncovered or covered SEMSs were used, and the type of SEMS was selected at the discretion of the attending endoscopists. SEMS lengths were 60 mm, 80 mm, 100 mm, and 120 mm. Uncovered and covered SEMSs were 22 mm and 20 mm in diameter, respectively.

**Fig. 1:**
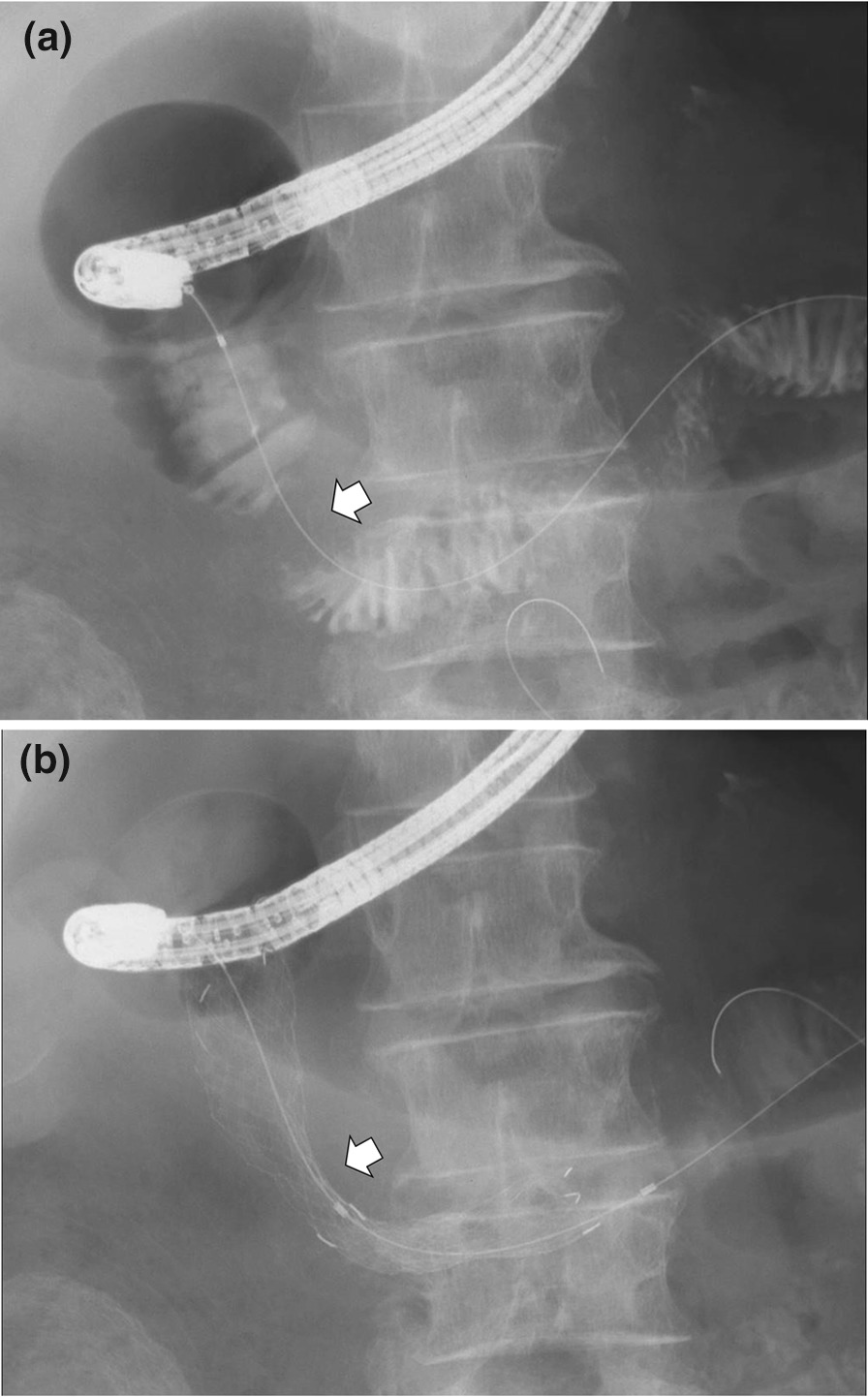
The process of duodenal stent placement. **a** A catheter with a guidewire is passed through the stenosis site (white arrow), and a water-soluble radiographic contrast medium is injected to determine the location and length of the stenosis site under fluoroscopic guidance. **b** A duodenal stent is deployed under endoscopic and fluoroscopic guidance. White arrow is showing stenosis cite

The endoscope was first used to approach the stenosis site, after which a guidewire and a catheter were passed through the site. A water-soluble radiographic contrast medium was injected through the catheter to determine the location and length of the stenosis site under fluoroscopic guidance (Fig. 1a). Then, SEMS was deployed at an adequate location under fluoroscopic guidance (Fig. 1b). Multiple SEMSs were used if the stenosis was long. In patients with coexisting mGOO and biliary obstruction prior to DSP, DSP for mGOO was performed first with transmural or percutaneous biliary drainage being conducted at a later date.

All patients were monitored in the hospital for at least 3 days after DSP. Patients without immediate AEs were allowed liquid diet intake 1–2 days after DSP. The patient’s diet was gradually changed according to their symptoms. If the patient developed any AEs, prompt treatment was provided at the discretion of the attending physicians. For example, in patients who developed biliary obstruction, antibiotics and biliary drainage was considered for acute cholangitis and obstructive jaundice. In patients who developed pancreatitis, intravenous therapy was initiated immediately after diagnosis.

### Data collection and definitions

**Fig. 2:**
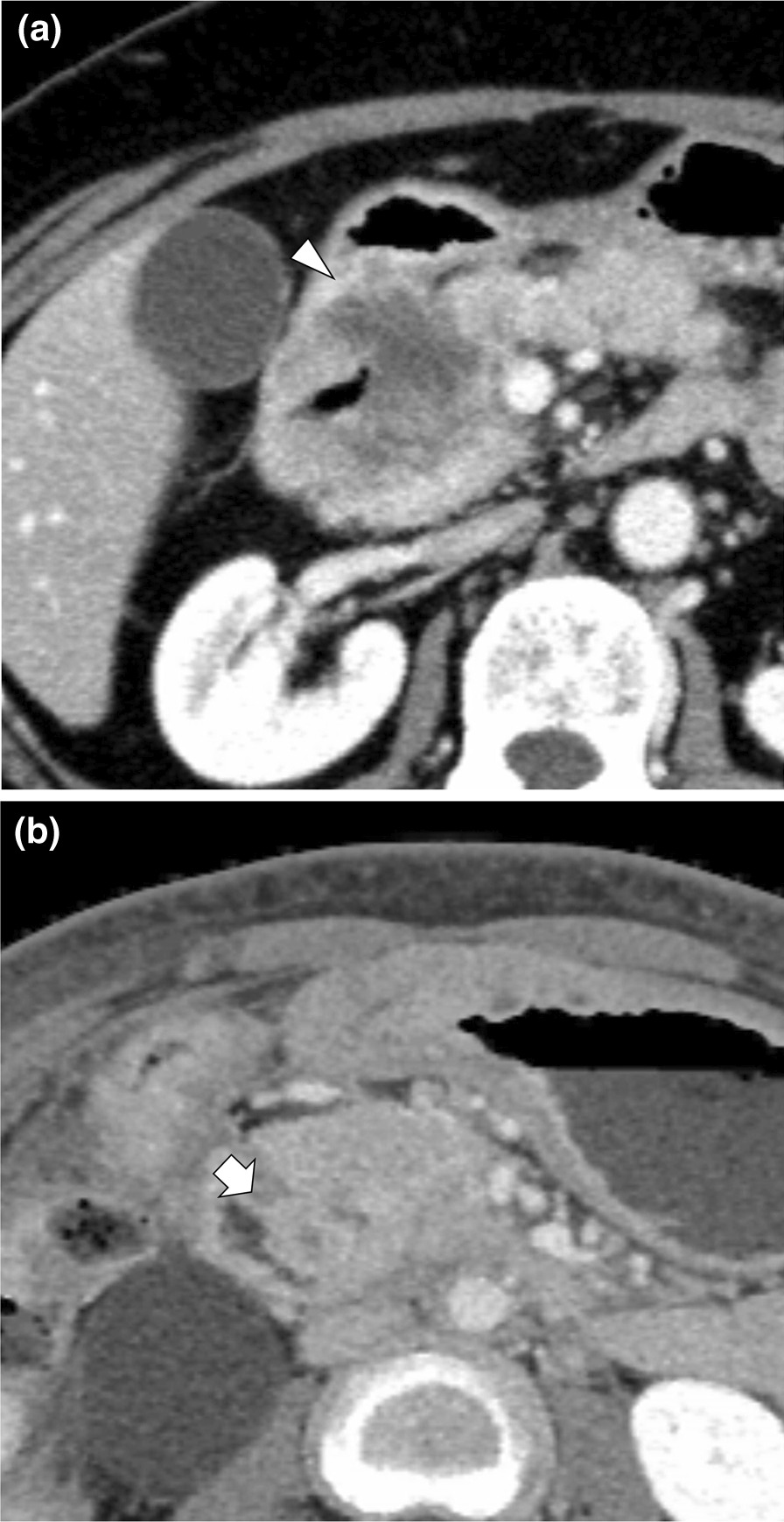
Computed tomography images showing tumor invasion to the main duodenal papilla **a** Pancreatic cancer is shown invading the main duodenal papilla directly (arrowhead). **b** Duodenal infiltration of the uterine cancer is shown invading the main duodenal papilla (arrow)

Data were collected from the patient medical records. The oral intake status was evaluated using the Gastric Outlet Obstruction Scoring System (GOOSS): 0, no oral intake; 1, liquids only; 2, soft solid diet; and 3, full solid diet [18]. The maximum diameters of the extrahepatic bile duct and main pancreatic duct were measured using axial CT images obtained within 1 month prior to DSP. Extrahepatic bile duct and pancreatic duct dilations was defined as > 10 mm and > 3 mm, respectively. Biliary stents were defined as those deployed across the MDP prior to DSP. The presence of tumor invasion to the MDP was evaluated by an experienced radiologist (K.A.) using a CT image obtained within 1 month before DSP (Fig. 2a, b). All identifying information was removed from the CT images, and the radiologist reviewing the images was also blinded to the information.

Technical success was defined as adequate DSP across the stenosis site as confirmed by endoscopy and fluoroscopy. Clinical success was defined as a GOOSS score of ≥ 2 and improvement in the GOOSS score after DSP.

BPAD was defined as biliary obstruction and/or pancreatitis that developed within 1 week of DSP in the descending duodenum. In other words, BPAD included biliary obstruction after DSP (BAD) and/or pancreatitis after DSP (PAD). The diagnosis of BAD was defined as per the following criteria: (1) bilirubin > 2 mg/dL or transaminases (Aspartate transaminase and Alanine aminotransferase > 1.5 times upper limit) after DSP; (2) appearance or worsening of biliary dilation after DSP. For patients with mGOO and biliary obstruction prior to DSP, BAD was defined as bilirubin or transaminases levels > 2 times greater than that prior to DSP. Severity of acute cholangitis following biliary obstruction was determined based on the Tokyo Guidelines 2018 [19]. Meanwhile, the diagnosis of PAD was defined as meeting at least two of the three following criteria: (1) abdominal pain consistent with acute pancreatitis; (2) serum lipase (or amylase) activity greater than three times the upper limit of normal; (3) characteristic findings of acute pancreatitis on contrast-enhanced CT or less commonly magnetic resonance imaging or transabdominal ultrasonography according to the Revision of the Atlanta Classification (RAC) criteria [20]. Severity of pancreatitis was also determined based on the RAC criteria [20]

### Statistical analysis

Continuous variables are presented as the median and range and were compared using the Mann–Whitney U-test. Categorical variables are presented as n values (%) and were compared using Fisher’s exact test. The risk factors for BPAD were analyzed using a logistic regression model for the following nine variables: age (< cut-off value vs. ≥ cut-off value), sex (male vs. female), tumor diagnosis (pancreatic cancer vs. others), absence of extrahepatic bile duct dilation (yes vs. no), absence of pancreatic duct dilation (yes vs. no), biliary stents (yes vs. no), tumor invasion to the MDP (yes vs. no), location of duodenal stents (1st to 3rd vs. others), and type of duodenal stent (covered vs. uncovered). The cut-off values of continuous variables were decided based on receiver operating characteristic curves and calculations using the Youden index (sensitivity + specificity–1). The factors with substantial impact (*P* < 0.2) in the univariate analysis were subsequently evaluated in a multivariate analysis. A *P*-value of < 0.05 was considered statistically significant for all tests. All statistical analyses were performed using EZR (Saitama Medical Center, Jichi Medical University, Saitama, Japan), which is a graphical user interface for R (The R Foundation for Statistical Computing, Vienna, Austria). More precisely, it is a modified version of R Commander designed to add statistical functions frequently used in biostatistics [21].

## Results

### Patient characteristics

**Fig. 3:**
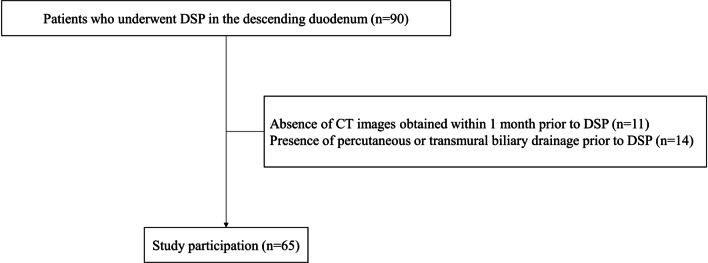
Ninety consecutive patients who underwent SEMS placement in the descending duodenum were enrolled; however, 11 and 14 patients were excluded because of absence of CT images obtained within 1 month prior to DSP and the presence of percutaneous or transmural biliary drainage prior to DSP, respectively. Therefore, the data of 65 patients were included in the analysis

Ninety consecutive patients who underwent SEMS placement in the descending duodenum were enrolled. Among these, 11 and 14 patients were excluded because of absence of CT images obtained within 1 month prior to DSP and the presence of percutaneous or transmural biliary drainage prior to DSP, respectively. Finally, the data of 65 patients were included in the analysis (Fig. 3) (Additional file 1).

Patient characteristics are shown in Table 1. Technical success and clinical success were achieved in 65 (100%) and 53 patients (82%), respectively. Of the patients in whom clinical success was not achieved, five exhibited no improvement in their GOOSS scores and seven did not achieve a GOOSS score ≥ 2, although their GOOSS scores had improved.

**Table 1:** Patient characteristics

Age (median, year) [range]	68 [40–91]
Sex (male/female)	38/27
ECOG performance status score (0/1/2/3/4)	4/23/23/13/2
Previous history (cholangitis/chronic pancreatitis/post-ERCP pancreatitis)	34/1/3
Primary cancer (pancreatic cancer/biliary tract cancer/gastric cancer/gynecologic cancer/duodenal cancer/others)	37/9/4/4/3/8
Gastric outlet obstruction scoring system score (0/1/2)	22/35/8
Extrahepatic bile duct diameter (median, mm) [range]	10 [2–24]
Pancreatic duct diameter (median, mm) [range]	4 [1–10]
Serum bilirubin (median, mg/dL) [range]	0.7 [0.1–8.4]
Serum aspartate transaminase (median, IU/L) [range]	32 [11–331]
Serum alanine aminotransferase (median, IU/L) [range]	25 [6–221]
Biliary stents (yes/no)	34/31
Type of stent (metallic stent/plastic stent)	32/2
Tumor invasion to the major duodenal papilla (yes/no)	42/23
*Duodenal stent*	
Location of duodenum stents (only 2nd part/1st part-2nd part/2nd part-3rd part/1st part-3rd part)	2/28/19/16
Length (6–9 cm/10 cm/12 cm/multiple stents)	16/21/26/2
Type (covered/uncovered)	6/59

Data are presented as n unless otherwise noted

*ECOG* eastern cooperative oncology group, *ERCP* endoscopic retrograde cholangiopancreatography

Eight patients had coexisting mGOO and biliary obstruction before DSP; Seven had transmural biliary drainage and one had percutaneous biliary drainage after DSP.

### Biliary obstruction and/or pancreatitis after duodenal stent placement

BPAD developed in 12 patients (18%), with BAD occurring in eight patients, PAD in two patients, and both BAD and PAD in two patients.

The median time of onset of BAD and PAD from DSP was 2 days (range; 1–7 days) and 0 days (range 0–1 days), respectively. In addition, in patients with both BAD and PAD, PAD developed first. The median time of onset of PAD was significantly shorter than that of BAD (*P* < 0.01).

All patients with BAD also had acute cholangitis. Severity grading of acute cholangitis was mild in two patients, moderate in six patients, and severe in two patients. Four patients with biliary obstruction were considered for biliary drainage, but this did not be performed due to the advanced stage of the cancer and poor general condition. As a result, they were treated with antibiotics only. Six patients underwent biliary drainage; transmural biliary drainage was performed in three patients and percutaneous biliary drainage was performed in three patients.

Meanwhile, severity grading of acute pancreatitis was mild in all four patients. There were no acute necrotic pancreatitis. All four patients improved their PAD with intravenous therapy and did not require intensive care or interventional therapy; however, two patients with both BAD and PAD required additional treatment for BAD.

Multivariate analysis revealed that female sex (odds ratio: 9.2, 95% confidence interval: 1.4–58.6, *P* = 0.02), absence of biliary stents (odds ratio: 12.9, 95% confidence interval: 1.8–90.2, *P* = 0.01), and tumor invasion to the MDP (odds ratio: 25.8, 95% confidence interval 2.0–340.0, *P* = 0.01) were independent risk factors for BPAD (Table 2).

**Table 2:** Results of the univariate and multivariate analyses of risk factors for biliary obstruction and/or pancreatitis after DSP in the descending duodenum

	n	Univariate analysis		Multivariate analysis	
		OR (95% CI)	*P*-value	OR (95% CI)	*P*-value
Age (years)					
≤ 69	40	2.13 (0.51–8.78)	0.30		
> 69	25	1			
Sex					
Female	27	3.58 (0.95–13.50)	0.06	9.16 (1.43–58.60)	0.02*
Male	38	1		1	
Tumor diagnosis					
Pancreatic cancer	37	0.71 (0.20–2.49)	0.59		
Other	28	1			
Extrahepatic bile duct dilatation					
Yes	21	1.65 (0.46–5.99)	0.45		
No	44	1			
Pancreatic duct dilation					
No	27	2.31 (0.65–8.27)	0.20		
Yes	38	1			
Biliary stents					
No	31	7.62 (1.52–38.30)	0.01*	12.90 (1.84–90.20)	0.01*
Yes	34	1		1	
Tumor invasion to the major duodenal papilla					
Yes	42	7.81 (0.94–64.90)	0.06	25.80 (1.96–340.00)	0.01*
No	23	1		1	
Position of duodenum covered by duodenal stents					
1st-3rd	16	4.30 (1.14–16.20)	0.03*	2.50 (0.40–15.70)	0.33
Others	49			1	
Types of duodenal stent					
Covered	6	0.87 (0.09–8.24)	0.91		
Uncovered	59	1			

*DSP* duodenal stent placement, *OR* odds ratio, *CI* confidence interval

*P* < 0.05^*^

Multivariate analysis revealed that independent risk factors for BAD were absence of biliary stents (odds ratio: 8.5, 95% confidence interval: 1.5–47.3, *P* = 0.01), and tumor invasion to the MDP (odds ratio: 10.1, 95% confidence interval 1.1–91.9, *P* = 0.04) (Table 3). However, statistical analysis of risk factors for PAD was not possible due to the small number of PAD cases. Patients with PAD are summarized in Table 4. All patients with PAD were female, had non-pancreatic cancer, no biliary stents, and tumor invasion of the MDP.

**Table 3:** Results of the univariate and multivariate analyses of risk factors for biliary obstruction after DSP in the descending duodenum

	n	Univariate analysis		Multivariate analysis	
		OR (95% CI)	*P*-value	OR (95% CI)	*P*-value
Age (years)					
≤ 64	22	0.44 (0.08–2.26)	0.32		
> 64	43	1			
Sex					
Female	27	2.43 (0.61–9.63)	0.21		
Male	38	1			
Tumor diagnosis					
Pancreatic cancer	37	1.16 (0.29–4.58)	0.83		
Other	28	1			
Extrahepatic bile duct dilatation					
Yes	21	0.89 (0.20–3.81)	0.87		
No	44	1			
Biliary stents					
No	31	5.57 (1.08–28.70)	0.04*	8.54 (1.54–47.30)	0.01*
Yes	34	1		1	
Tumor invasion to the major duodenal papilla					
Yes	42	6.00 (0.71–50.70)	0.10	10.10 (1.10–91.90)	0.04*
No	23	1		1	
Position of duodenum covered by duodenal stents					
1st-3rd	16	2.39 (0.58–9.86)	0.23		
Others	49	1			
Types of duodenal stent					
Covered	6	1.11 (0.12–10.70)	0.93		
Uncovered	59	1			

*DSP* duodenal stent placement, *OR* odds ratio, *CI* confidence interval

*P* < 0.05^*^

**Table 4:** Pacnreatitis after DSP in the descending duodenum

Age/ Sex	Primary cancer	Pancreatic duct diameter	Biliary stents	Tumor invasion to the MDP	Duodenal stents	Complication of BAD	Position of duodenal stent	Sevierity of pancreatitis	Treatment
53/ F	gynecologic cancer	8 mm	No	Yes	Two 12-cm UCSEMS	No	1st-3rd	Mild	Intravenous therapy
78/ F	unknown primary	1 mm	No	Yes	One 12-cm UCSEMS	Yes	2nd-3rd	Mild	Intravenous therapy
68/ F	gynecologic cancer	4 mm	No	Yes	One 12-cm UCSEMS	No	1st-3rd	Mild	Intravenous therapy
69/ F	gynecologic cancer	4 mm	No	Yes	One 12-cm UCSEMS	Yes	1st-3rd	Mild	Intravenous therapy

*DSP* duodenal stent placement, *MDP* major duodenal papilla, *BAD* biliary obstruction after DSP

### Early adverse events other than biliary obstruction and pancreatitis

There was stent migration in one patient. The migrated SEMS was removed, and a new one was deployed, after which the patient’s symptoms improved without any further stent migration.

## Discussion

In this retrospective study, BPAD occurred in 18% (12/65) of patients with DSP in the descending duodenum. We identified female sex, tumor invasion to the MDP, and absence of biliary stents as risk factors for BPAD. In addition, we also found that tumor invasion to the MDP and absence of biliary stents were identified as risk factors for BAD.

Several studies have reported the development of BPAD [11, 16, 17]. SEMSs placed over the MDP can obstruct the flow of the bile and pancreatic ducts, resulting in BPAD. Thus, ideally, SEMS placement over the MDP should be avoided. However, this is often difficult for the following reasons. In cases with duodenal stenosis on the oral side of the MDP, the endoscope cannot approach the MDP, resulting in failure to confirm the location of the MDP. Also, in cases with duodenal stenosis on or near the MDP, SEMSs must be placed over the MDP for the treatment of mGOO symptoms, even though the MDP can be confirmed endoscopically. As SEMS placement over the MDP often cannot be avoided, endoscopists should be aware of the risk of BPAD when they place SEMSs in the descending duodenum. Therefore, knowledge regarding the incidence of and risk factors for BPAD is useful in clinical practice (Additional file 1).

In the present study, three main risk factors for BPAD were observed: female sex, tumor invasion to the MDP, and absence of biliary stents. First, in this study female sex was a risk factor for BPAD, but not for BAD; however, all patients with PAD were female, which may indicate that the female sex may be predisposed to PAD despite statistical analysis not being performed. This result may be related to the predisposition of females to post-ERCP pancreatitis [22, 23]. Female patients may be more responsive to MDP-related irritation than male patients. Second, in patients with tumor invasion to the MDP, the biliary or pancreatic duct may exhibit a pre-obstructive state, and compression from the SEMS may easily trigger symptoms. This mechanism may be similar to that of acute cholecystitis after biliary stent. Several previous studies have indicated that tumor involvement in the orifice of the cystic duct is a risk factor for cholecystitis after biliary stent [24–26]. Third, biliary stents may prevent BPAD. Generally, the biliary stent is 1 cm or more in the duodenal lumen. Therefore, when SMESs are deployed in the descending duodenum, the biliary stent may act as a prop, and may relieve MDP compression. However, previous studies have reported that DSP is a risk factor for the dysfunction of biliary stents deployed over the MDP; thus, transmural biliary drainage may be preferred over transpapillary biliary drainage for patients with mGOO accompanied by biliary obstruction [27, 28]. In addition, transpapillary biliary drainage for a patient with duodenal stent is challenging. Thus, biliary stents may not be deployed for the prevention of BPAD. This study found three main risk factors for BPAD; however, evidence to support our results remains insufficient. Thus, further research will be required to validate our results.

In our study, the median time of onset of PAD was significantly shorter than that of BAD (0 day vs. 2 days). Additionally, in patients with both BAD and PAD, PAD developed first followed by BAD. This may be related to the time course from bile duct and pancreatic duct obstruction to symptoms. In an experiment using a model of pancreatic duct obstruction in rats, serum amylase activity increased five-fold within 1 h of pancreatic duct obstruction and, the degree of edema continued to increase over the 6 h following pancreatic duct obstruction [29]. However, in an experiment using a model of biliary obstruction in rats, the serum bilirubin 5 days after interruption of bile flow was elevated [30]. Similarly, there may be a difference in the onset time of BAD and PAD.

The common alternative treatment for DSP is surgical gastrojejunostomy, but patients with mGOO are often in poor general condition and unsuitable for surgical gastrojejunostomy. Recent studies have demonstrated the efficacy of endoscopic ultrasonography-guided gastroenterostomy (EUS-GE) in the management of mGOO [31]. In one retrospective study, EUS-GE had a higher rate of initial clinical success and lower rate of stent failure requiring repeat intervention than DSP [32]. In addition, in EUS-GE, there is no risk for biliary obstruction or pancreatitis because the SEMS is not placed over the MDP. Thus, EUS-GE may be suitable for patients with mGOO with a high risk of BPAD. However, EUS-GE poses many challenges, including the lack of dedicated devices and non-availability at many hospitals in Japan. Currently, DSP is the standard for mGOO treatment, even for patients at high risk of BPAD. Therefore, risk stratification for BPAD is required to inform patients in detail prior to DSP.

This study had several limitations. First, there may have been unintentional bias because of its retrospective study design. Second, the sample size was too small given the single-center nature of the study, Third, in the present study, both covered and uncovered SEMSs were used for DSP, that present with different AEs. Covered SEMS have been reported to have a higher incidence of stent migration, but a lower incidence of stent re-obstruction, compared with uncovered SEMS [10]. However, a multicenter randomized prospective study showed there were no difference in the incident of jaundice and/or cholangitis and pancreatitis between covered and uncovered SEMSs [33]. Therefore, in the present study, a dataset containing both covered and uncovered SEMSs was used. Forth, there is insufficient information regarding the patency of biliary stents. Furthermore, many of the biliary stents were deployed in the previous hospital, so it was not possible to obtain information regarding stent patency. However, common causes of biliary stents include tumor ingrowth, tumor overgrowth, biliary sludge, and food impaction. Conversely, BAD is caused by compression of the MDP due to DSP. Therefore, the stent patency after DSP may differ from that of a typical biliary stent because of a different cause of stent occlusion. Fifth, risk for PAD could not be evaluated due to the small number of PAD cases. Thus, larger clinical trials are required to assess risk factors for BAD and PAD separately and simultaneously.

In conclusion, our findings indicated that the incidence of BPAD was non-negligible among patients who underwent DSP in the descending duodenum. Female sex, absence of biliary stents, and tumor invasion to the MDP were identified as potential risk factors for BPAD. In addition, absence of biliary stents, and tumor invasion to the MDP were identified as potential risk factors for BAD. Risk stratification can allow endoscopists to better identify patients who are at significant risk and permit detailed informed consent and high-risk groups may be offered non-DSP treatment in the future.

## Supplementary Information

**Additional file 1.** Database of this study.

## Data Availability

All data generated or analyzed during this study are included in this published article and its Additional files.

## Abbreviations

AE: Adverse event
BPAD: Biliary obstruction and/or pancreatitis after duodenal stent placement
BAD: Biliary obstruction after duodenal stent placement
CT: Computed tomography
DSP: Duodenal stent placement
ECOG: Eastern cooperative oncology group
ERCP: Endoscopic retrograde cholangiopancreatography
EUS-BD: Endoscopic ultrasound-guided biliary drainage
EUS-GE: Endoscopic ultrasonography-guided gastroenterostomy
GOOSS: Gastric outlet obstruction scoring system
IRB: Institutional review board
MDP: Major duodenal papilla
mGOO: Malignant gastric outlet obstruction
PAD: Pancreatitis after duodenal stent placement
PTBD: Percutaneous transhepatic biliary drainage
SEMS: Self-expandable metallic stents
